# Cortical Alpha Activity in Schizoaffective Patients

**Published:** 2017-01

**Authors:** Mahdi Moeini, Ali Khaleghi, Mohammad Reza Mohammadi, Hadi Zarafshan, Rachel L. Fazio, Hamid Majidi

**Affiliations:** 1 Department of Psychiatry, School of Medicine, Isfahan University of Medical Sciences, Isfahan, Iran.; 2 Psychiatry & Psychology Research Center, Tehran University of Medical Sciences, Tehran, Iran.; 3 Carter Psychology Center, Bradenton, Florida, USA.; 4 Technical and Vocational University, Hamedan, Iran.

**Keywords:** *Alpha Activity*, *Electroencephalogram*, *Schizoaffective Disorder*, *Serotonin*

## Abstract

**Objective: **Electrophysiological studies have identified abnormal oscillatory activities in the cerebral cortex in schizophrenia and mood disorders. Biological and pathophysiological evidence suggests specific deficits in serotonin (5-HT) receptor function in schizoaffective disorder (SA), a clinical syndrome with characteristics of both schizophrenia and bipolar disorder. This study investigated alpha oscillations in patients with SA.

**Method:** Electroencephalography was used to measure ongoing and evoked alpha oscillations in 38 adults meeting Diagnostic and Statistical Manual of Mental Disorders–Fourth Edition (DSM–IV) criteria for SA, and in 39 healthy controls.

**Results:** Spontaneous alpha power of the participants with SA was significantly lower than that of healthy participants [F (1, 75) = 8.81, P < 0.01]. Evoked alpha activity was also decreased in SA compared to controls [F (1, 75) = 5.67, P = 0.025].

**Conclusion**: A strong reduction of alpha power in the posterior regions may reflect abnormality in the thalamocortical circuits. It is shown that hypoxia and reduced cerebral blood flow is associated with reduced alpha activity among different regions of the brain. Therefore, it can be concluded that greatly decreased alpha activity, particularly in centro-parietal and occipital regions, is related to SA symptoms such as hallucinations.

During the past few decades, the concept of oscillatory brain dynamics has attracted much attention in neuroscience research. Since the 1990s, applications of measurement of oscillatory activity have grown rapidly in clinical pathology. There are many studies in this field and our research group commenced research on pathological brain oscillations in bipolar disorder (1-3), attention-deficit/hyperactivity disorder (4, 5), and schizoaffective disorder (6). Schizoaffective disorder (SA) is a severe, chronic psychiatric disorder that consists of symptoms of schizophrenia and affective disorders concurrently (7), with a lifetime prevalence of 0.3% (7, 8) which is more common in females than males (9). Genetic findings suggest GABA-A receptor dysfunction influences the duration of inhibitory post synaptic current onto pyramidal cells in SA (especially in bipolar type) (10-12). Due to the association between cortical gamma oscillations (30-80 Hz) and excitatory-inhibitory activity generated between GABA interneuron cell assemblies and reciprocally connected glutamatergic cells (13, 14), a recent magnetoencephalography (MEG) study (15) investigated gamma activity in schizoaffective bipolar disorder. The authors reported an increased gamma power in remitted schizoaffective bipolar disorder, which represents an abnormalities in the cortical excitatory-inhibitory balance.

Furthermore, some biological and neuropathological evidence suggests specific deficits in serotonin (5-HT) receptor function in SA (16, 17). Serotonin plays the role of neuromodulator/neurotransmitter in the central nervous system (CNS). Given the extensive innervations of the serotonergic system within the CNS and the brain, the 5-HT system is involved in many functions, and it is the target of numerous drugs used to treat psychiatric and brain disorders. Among the cortical areas, the frontal lobe is the richest region in 5-HT receptors and serotonergic terminals. 

Desired levels of 5-HT in the prefrontal and frontal cortices are necessary for behavioral inhibition and modulation of attention in humans.

5-HT axons have a large impact on generation of action potentials by establishing axo-axonic contacts; previous studies suggested a link between 5-HT levels and the magnitude of alpha oscillations from electroencephalography (EEG) (18).

However, the observations of 5-HT levels and alpha activity are inconsistent in SA. Some studies (16, 17) reported downregulation or upregulation of cortical serotonergic activity in SA that may affect the alpha oscillations, resulting in higher or lower alpha activity in the cortex. 

To the best of our knowledge, few studies have analyzed the spontaneous EEG and visual evoked potentials of SA patients. Although spontaneous EEG of SA has been studied before, no study investigated the spontaneous EEG of SA patients with acute episodes. Therefore, the aim of this study was to analyze the resting state EEG alpha activity of SA patients with closed and open eyes. We hope the results of this study provide some insight into the mechanisms causing the disorder and also clarify the previously mentioned contradictory findings.

## Materials and Methods

***Participants***

For the study group, our sampling frame was the list of all patients referred to Razi psychiatric hospital (Tehran, Iran) and hospitalized in a five year period. Of the 131 adult patients with SA who were hospitalized in these five years, 76 patients were selected by a random sampling method. Then, the patients were evaluated considering the inclusion and exclusion criteria. All participants in this group met Diagnostic and Statistical Manual of Mental Disorders (4th ed.; DSM–IV-TR; American Psychiatric Association, APA, 2000) diagnostic criteria for SA as determined by an experienced psychiatrist; all had at least one episode in the past and were experiencing symptoms at the time of the study. Eight patients were excluded after evaluation due to psychiatric comorbidity, another eight were excluded due to substance abuse, seven were excluded due to epileptic seizures or severe head injuries, two were excluded due to major medical illness, and six were excluded due to their or their family’s unwillingness to participate in the study. Seven patients had poor cooperation during the signal recording, so they were excluded from the study. This resulted in the SA group consisting of 23 men and 15 women, with a mean age of 35.40 years (SD = 12.61). Based on the psychiatrist’s diagnosis, 35 of the SA patients met criteria for the bipolar type, whereas three were diagnosed with depressed type.

The control group was selected randomly from the available people without SA disorder. Fifty-four individuals were selected and interviewed by a psychiatrist, six were excluded because of meeting the criteria for psychiatric diagnosis, four were excluded due to a history of neurological disorders, two were reluctant to participate in the study, and three were excluded due to poor cooperation during EEG recording, resulting in 39 healthy control participants. This group contained 22 men and 17 women, with an average age of 34.70 (SD = 14.00). There was no significant difference between the SA group and the control group in age or gender composition.

***Apparatus***

A 32 channel recording system was employed (Micromed Brain Quick 98 QEEG device). Electrodes were placed at standard locations based on 10-20 international system. Electrodes were placed at recording positions Fz, Cz, Pz, Fpz, Oz, C3, T3, C4, T4, Fp1, Fp2, F3, F4, F7, F8, P3, P4, T5, T6, O1 and O2. Clip electrodes on the left and right earlobes were used as reference. Eye movement was recorded by two electrodes that were placed below and above the right eye.

***Experimental procedures***

The experiment was performed at usual ambient light levels and in a quiet room to minimize sensory interference. Participants were seated comfortably on a chair during EEG recording and were asked to be immobile to minimize muscular artifacts. The first two minutes of data recorded were discarded to allow participants to adapt to the environment. EEG was recorded in band limits of 0.4-70 Hz and digitized at a sampling rate of 512 Hz. First, spontaneous EEG of the individual was recorded for 8-10 min in open- and closed-eyes conditions. Then, the participants were subjected to white light stimulations during EEG recording. The stimulation was delivered by an array of LEDs (17 × 4 LEDs) with white light and 10 cd/cm2 luminance. The light stimulator unit was held approximately 35 cm from the eyes. The duration of each stimulation was 1000 ms with inter-stimulus of 500 ms. Multiple blocks (four to six) of thirty 1000-ms white light stimulations were delivered to the participants. This research was approved by the Ethical Committee and Institutional Review Boards at Tehran University of Medical Sciences. All participants or their family read and signed an informed consent to be involved in the study.

***EEG Analysis***

**A. Spontaneous EEG activity**

The spontaneous or resting-state EEG analysis was performed for open-eyes and closed-eyes conditions separately. The analysis was conducted on the sixty one-second epochs that were selected by an experienced technician (by visual inspection) to be artifact-free after noise reduction, using a Butterworth IIR band-pass filter and a notch filter of 50 Hz. Then, Welch technique, a FFT (Fast Fourier Transform)-based method, was applied to perform the power spectrum analysis and to determine the alpha oscillations (8-13 Hz) in EEG. 

**B. Visual Evoked Oscillations**

After noise reduction, the trials with artifacts were identified and removed. On average, 80 artifact-free trials (with duration of 1000 ms) were selected and after FFT application to obtain the power spectrum for each trial, they were averaged for each subject. Finally, the averaged spectrum was filtered to determine the alpha oscillations (8-13 Hz).

***Statistical Analysis***

Statistical analysis was performed by SPSS (Version 21). Age and sex data between groups was evaluated by a two-sample t-test. A repeated measures analysis of variance (ANOVA) was used to determine the statistical significance of alpha activities over conditions, regions and among patients and controls. Two separate repeated measures were used for both spontaneous and visual oscillations EEG: the between-participants factor as healthy controls, and SZ patients. The within-subject factors were condition (open-eyes and closed-eyes), brain regions including sagittality (anterior, posterior) and laterality (left, right and midline). However, there was no closed-eyes condition in visual evoked EEG, and the within-subject factor only included brain regions (sagittality and laterality). If necessary, Bonferroni correction was applied for multiple comparisons. Furthermore, multivariate analysis of variance (MANOVA) was used to analyze the between-subject effects. Greenhouse-Geisser estimates were reported when the assumption of sphericity was broken. The level of significance was set at P < 0.05.

## Results

**Figure1:**
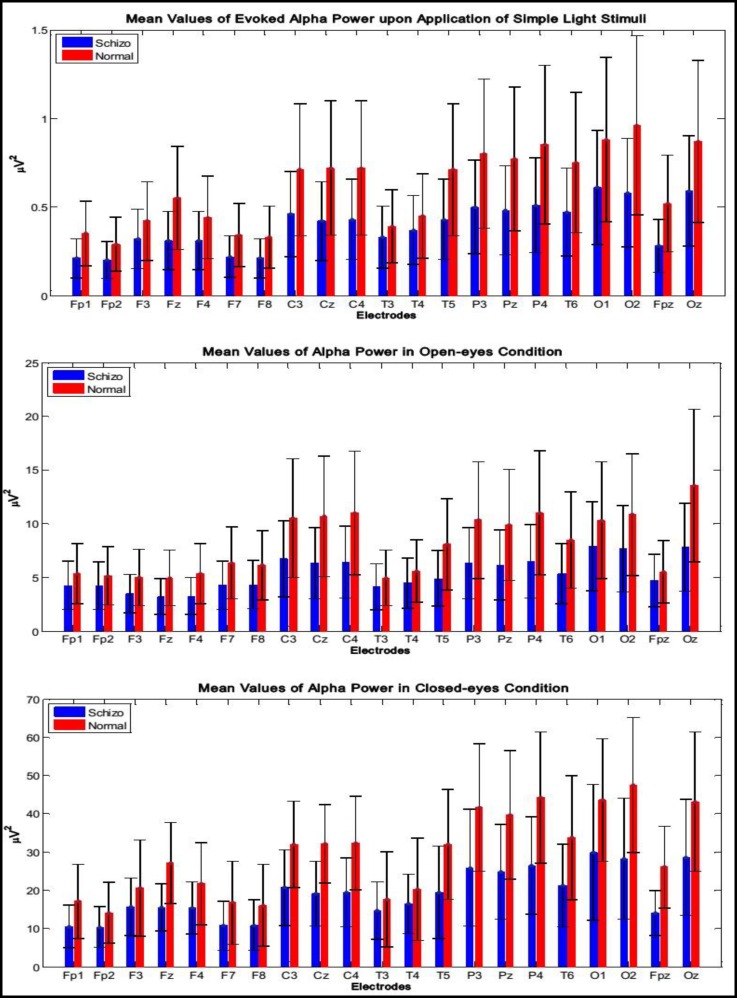
Mean Values of Evoked Alpha Power of 39 Healthy and 38 Schizoaffective Participants (Top Panel). Mean Values of Alpha Power of 39 Healthy and 38 Schizoaffective Participants in Open-Eyes (Middle Panel) and Closed-Eyes (Bottom Panel) Conditions

Demographic and other descriptive characteristics of the two groups are presented in Table 1. No significant differences were observed for baseline characteristics. Figure 1 displays the mean values of alpha power in all 21 channels in 38 healthy participants and 39 SA patients for the open-eyes and closed-eyes conditions. The mean values of evoked alpha power are also displayed in this figure. It can be seen that the alpha power spectrum of the healthy controls is considerably higher than that of SA patients; it was as high as about 47 μV2 in O2 electrode for the control group, whereas the alpha power spectrum of the SA patients reached about 28 μV2 in O2 electrode or 30 μV2 in O1 electrode.

***Statistical Observations of Spontaneous EEG activity***

The repeated measures ANOVA of the pattern of alpha activity revealed significant effects for sagittality [F (1, 75) = 60.58, p < 0.001], conditions (open- and closed-eyes) [F (1, 75) = 28.12, p < 0.001], sagittality × laterality interaction [F (2,150) = 3.17, p = 0.05], sagittality × laterality × group interaction [F (2,150) = 3.76, p = 0.029], condition × group interaction [F (1, 75) = 4.35, p = 0.018] and condition × region interaction [F (2,150) = 16.34, p < 0.01]. There were no significant differences for laterality [F (2,150) = 1.76, p = 0.18], laterality × group interaction [F (2,150) = 1.97, p = 0.14] and sagittality × group interaction [F (1, 75) = 3.09, p = 0.09]. 

Tests of between-subjects effects revealed a significant effect for group [F (1, 75) = 8.81, p < 0.01]. Based on the results of multivariate ANOVA, using Pillai’s trace, there was a significant effect for group (V = 0.54, F (1, 75) = 4.38, p < 0.01). Tests of between-subjects effects and pairwise comparisons (Table 2) showed that the alpha power was significantly reduced in the patient group compared to the normal group across all locations of the brain.

***Statistical Observations of Visual Evoked Oscillations***

**Figure2:**
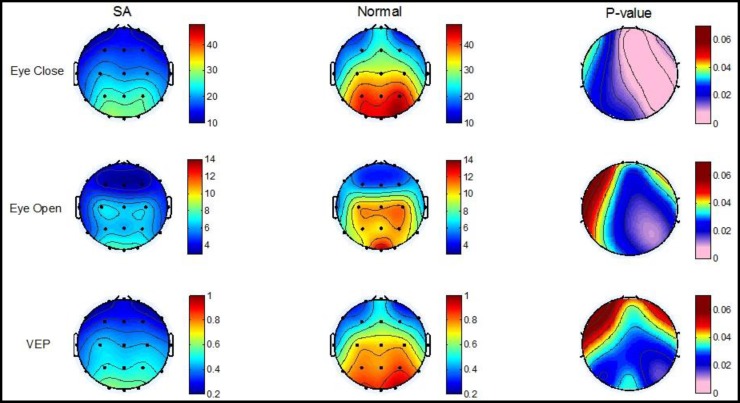
Grand Averages of Alpha Activity of 39 Healthy and 38 Schizoaffective Participants of Electroencephalograms (EEGs) Recorded in Closed-Eyes (Top), Open-Eyes (middle) and Visual Evoked Responses (Bottom) along with Statistical Observations in Each Condition. (SA = Schizoaffective)

The repeated measures ANOVA of alpha responses disclosed significant effects for sagittality [F (1, 75) = 18.80, p < 0.001] and sagittality × laterality × group interaction [F (2,150) = 4.53, p = 0.043]. On the other hand, no significant differences were observed for laterality [F (1, 75) = 2.06, p = 0.162], sagittality × group interaction [F (1, 75) = 1.41, p = 0.246], laterality × group interaction [F (2,150) = 1.96, p = 0.172], or sagittality × laterality interaction [F (1, 75) = 16.47, p = 0.052]. ANOVA of the alpha activity (Table 3) revealed significant differences between groups [F (1, 75) = 5.67, p = 0.025]. The results of multivariate ANOVA indicated that the visual evoked alpha activity of SA patients was significantly lower than that of the healthy participants (p < 0.01). Figure 2 displays the grand average of alpha frequency power spectrum in the two groups as a topographic comparison. Highly lower alpha activity in all brain regions was apparent in SA patients regardless of recording conditions.

## Discussion

In this study, we assessed the neurophysiological evidence for relations between abnormalities in the alpha frequency power spectrum of the brain’s electrical activity and symptoms in schizoaffective patients.

We sought differences in alpha activity between EEGs acquired from adults with and without SA in different conditions.

**Table1:** Baseline Characteristics of Patients with Schizoaffective Disorder and Normal Participants

**Parameters**	**SA (n = 38)**	**HC (n = 39)**	**P-value**	**t**
Age	44.7±14	42.4±12.6	0.134	1.569
Gender (male, female)	23, 15	22, 17	0.653	-0.451
Lifetime psychiatric hospitalization	3.86±2.13	N/A		
Age of first psychiatric hospitalization	27.46±5.81	N/A		
Antipsychotics	37	0		
Handedness	Right (33), Left (4), Ambidextrous (1)	Right (34), Left (5)		

**Table2:** Pairwise Comparison of Spontaneous EEG between Schizoaffective Patients and Healthy Participants Using Multivariate ANOVA

**Dependent ** **Variable**	**GROUP (I)**	**GROUP (J)**	**Mean Difference (I-J)**	**SE**	**p.** ^a^	**95% Confidence ** **Interval for ** **Difference**		**Observed ** **Power**
						**Lower ** **Bound**	**Upper ** **Bound**	
Left anterior	Patient	normal	-8.104*	3.506	0.029	-15.298	-.910	0.606
Right anterior	Patient	normal	-9.627*	3.466	0.010	-16.740	-2.515	0.763
Midline anterior	Patient	normal	-12.248*	3.213	0.001	-18.839	-5.656	0.957
Left posterior	Patient	normal	-14.715*	5.999	0.021	-27.024	-2.406	0.657
Right posterior	Patient	normal	-18.233*	5.733	0.004	-29.996	-6.470	0.866
Midline posterior	Patient	normal	-14.755*	5.818	0.017	-26.692	-2.819	0.686

Note. ANOVA = analysis of variance.

* The mean difference is significant at the.05 level. ^a^ Adjustment for multiple comparisons: Bonferroni.

**Table3:** Pairwise Comparison of Visual Evoked Alpha Activity between Schizoaffective Patients and Healthy Participants Using Multivariate ANOVA

**Dependent ** **Variable**	**GROUP (I)**	**GROUP (J)**	**Mean Difference (I-J)**	**SE**	**p.** ^a^	**95% Confidence ** **Interval for ** **Difference**		**Observed ** **Power**
						**Lower ** **bound**	**Upper ** **bound**	
Left anterior	Patient	normal	-0.152	0.025	0.067	0.253	0.351	0.394
Right anterior	Patient	normal	-0.210	0.105	0.052	-0.363	0.057	0.451
Midline anterior	Patient	normal	-0.271^*^	0.135	0.038	-0.562	-0.020	0.542
Left posterior	Patient	normal	-0.285^*^	0.142	0.023	-0.614	-0.044	0.648
Right posterior	Patient	normal	-0.360^*^	0.180	0.016	-0.783	-0.063	0.684
Midline posterior	Patient	normal	-0.289^*^	0.144	0.034	-0.604	-0.026	0.563

The application of spectral analysis to EEG can provide some important information about cerebral cortex abnormalities in neuropsychiatric disorders. Alpha frequency band is a very important frequency range that is impaired in most psychiatric disorders. Unbalanced alpha activity in the brain can represent sub-optimal cognitive performance and behavioral or mood disorders or other psychiatric symptoms. 

The literature includes just a few studies on EEG spectral analysis in SA patients with various approaches. Different from prior investigations, the present study focused on the alpha activity in visual evoked oscillations jointly with spontaneous EEG. In addition, the patients included in this study were experiencing at least their second episode of the disorder, and this made the analysis unique in the literature. Although our stimulation procedure was based on flash VEP (FVEP) patterns with low rate that produced transient visual evoked oscillations with minimal alpha activity, EEGs recorded in this condition may provide additional information about alpha responses at different levels of alpha generation in the brain.

Our results revealed a large reduction in alpha activity (50% to 70%) in SA patients compared to healthy controls, as found in a previous study (19).

This observation was consistent among all EEG recording conditions, which can result in increased reliability of the finding.

According to Başar (20), decreased ongoing activity within a specific frequency leads to reduction in the evoked responses within that frequency, which is in line with the observations in this study.

We also examined the alpha asymmetry during spontaneous EEG recording and found no significant differences in alpha asymmetry (or lateralization effects) between the two groups (this analysis and its results have not been reported here).

The finding of alpha reduction in the posterior regions of the brain may reflect abnormality in the thalamocortical network in SA. Furthermore, it was found that hypoxia and reduced cerebral blood flow is associated with reduced alpha activity among different regions of the brain (21). Therefore, it can be concluded that greatly decreased alpha activity, particularly in centro-parietal and occipital regions, is related to SA symptoms such as hallucinations.

## Conclusion

In summary, greatly decreased alpha activity, particularly in centro-parietal and occipital regions, is related to SA symptoms such as hallucinations. Therefore, in the future, we need relevant studies to investigate the electrophysiology and neurophysiology in the patients with schizoaffective disorders by designing appropriate visual tasks.

## Limitations

The major limitation of this study was that the patients were not drug-free and this could affect the obtained results. There were, however, a few evidences on the effect of antipsychotic drugs on EEG. For example, Schellenberg et al. (19), Jones et al. (22), Takahashi et al. (23), and Yamada et al. (24) reported no effects of typical and atypical antipsychotic treatment on the power spectral in EEG (except gamma oscillation; not relevant to this study). However, conducting a study by considering the different antipsychotic drugs at different ages would be useful in the future. Although most studies focused on dopamine levels and its effects on prefrontal cortical function (25-27), some researchers underlined the role of serotonergic system dysfunction in SA symptoms. Indeed, SA patients suffer from impulsivity, inattention, and reduced behavioral control, which suggests an upregulation of cortical serotonergic activity. Since the finding of abnormality in the anterior alpha power may be caused by abnormality in 5-HT levels, we aim to replicate this study by recording the EEG and 5-HT levels simultaneously in the future. 
